# Antimicrobial Sponge: A Polyvinyl Alcohol, Tannic Acid and Curcumin-Loaded Nanolignin Hydrogel Composite Scaffold

**DOI:** 10.3390/gels11030168

**Published:** 2025-02-26

**Authors:** Resmi Anand, Delphine Collard, Jean-Sébastien Thomann, David Duday

**Affiliations:** Environmental and Industrial Biotechnologies Unit, Luxembourg Institute of Science and Technology, L-4362 Esch-Sur-Alzette, Luxembourg; delphine.collard@list.lu (D.C.); jean-sebastien.thomann@list.lu (J.-S.T.)

**Keywords:** lignin nanoparticles, curcumin, drug delivery, encapsulation, antimicrobial

## Abstract

Materials with antimicrobial properties and high adsorption capabilities are crucial for managing exudate in post-surgical cases. However, achieving both properties simultaneously remains a challenge. In this study, we first synthesized curcumin-loaded organosolv lignin nanoparticles (Lig-Cur Nps) using a solvent-shifting approach in a continuous flow reactor. These Lig-Cur NPs were then dispersed in a polyvinyl alcohol (PVA) solution. The PVA-Lig-Cur NP colloidal suspension was further crosslinked with tannic acid (TA) through hydrogen bonding interactions. A simple freeze–thaw cycle of the PVA-Lig-Cur NP suspension with TA resulted in the formation of a stable gel, which was then lyophilized to fabricate the PVA-Lig-Cur-TA hydrogel scaffold. This scaffold features an interconnected microporous network with a swelling percentage of 800%, enabling the rapid adsorption of exudates. Its excellent properties and antimicrobial efficacy against *Staphylococcus aureus*, a bacterium commonly found on the skin, and *Pseudomonas aeruginosa* highlight its potential to effectively remove exudates while preventing bacterial colonization.

## 1. Introduction

The removal of exudate in post-surgical cases is crucial for effective wound healing and the prevention of post-surgical infections. Excessive fluid accumulations can lead to complications such as delayed healing, infection at the surgical site, or surgical wound dehiscence [1]. Therefore, effective management of exudate in post-surgical cases is essential for reducing complications and shortening the healing time for patients after surgery. Materials that provide both high adsorption capabilities and antimicrobial properties are essential for ensuring optimal wound environments. Traditional materials such as gauze have limited fluid absorption capacity [2]. In contrast, newly engineered materials, such as hydrogels, colloids, and polymer sponges, can absorb larger volumes of fluids. However, the long-term contact between wound dressings and tissue can lead to bacterial infection. Consequently, there is a growing interest in developing advanced materials that can effectively address these challenges [3].

Recent advancements in wound dressing materials have focused on creating multifunctional matrices. Curcumin, a natural polyphenolic bioactive compound known for its antimicrobial properties and wound-healing mechanism, has gained attention for its potential applications in wound management. Curcumin can act as a radical scavenging agent, reducing oxidative stress and inflammation at the wound site [4]. However, its poor solubility in water and low bioavailability have limited its efficacy in practical applications. Nanoparticle formulations of curcumin present a promising method to mitigate these drawbacks [5]. Meanwhile, organosolv lignin, a natural polyphenolic macromolecule, offers biocompatibility and can enhance the properties of composite materials. Lignin is recognized as a prominent natural antioxidant, possessing anti-inflammatory, UV-locking, antibacterial, and antifungal properties, which make it a suitable biobased carrier for curcumin. Gao et al. grafted curcumin to lignin through an esterification reaction prepared curcumin-grafted lignin nanoparticles and observed that the photothermal stability and biocompatibility of curcumin were effectively improved [6]. Alqahtani et al. reported the synthesis of crosslinked curcumin and encapsulated lignin nanoparticles using a modified phase separation method for oral drug delivery applications [7]. In another study, they demonstrated that wounded rats treated with curcumin-loaded lignin nanoparticles exhibited enhanced dermal wound closure compared to the control sample (an untreated wound) [8].

In the present study, we aimed to synthesize curcumin-loaded organosolv lignin nanoparticles and integrate them into a biocompatible and biodegradable polymer hydrogel matrix, developing a composite porous scaffold that exhibits strong antimicrobial activity and exceptional adsorption characteristics. Polyvinyl alcohol (PVA) is a water-soluble, semicrystalline, biocompatible, and biodegradable synthetic polymer that has been utilized in tissue engineering, wound dressing, and drug delivery applications [9,10]. The incorporation of lignin or curcumin into PVA scaffolds has been discussed in several recent publications [11,12]. For instance, Rejmontová et al. incorporated kraft lignin into a PVA scaffold to enhance antibacterial properties and biocompatibility [13]. Kang et al. reported that the addition of PVA increased the chemical and photostability of curcumin in aqueous solutions, thereby enhancing its activity in various antioxidant assay systems. Furthermore, Mahmud et al. reported the fabrication of a curcumin-loaded polyvinyl alcohol electrospun mat, which exhibited antibacterial activity against both Gram-positive and Gram-negative bacteria. These fibrous mats were crosslinked using heat and UV treatment [8]. While PVA can form a hydrogel through a single freeze–thaw cycle, it dissolves slowly when used alone. Therefore, crosslinking with a suitable crosslinking agent is necessary to enhance hydrogel stability. In this study, we crosslinked PVA with tannic acid, a natural polyphenolic crosslinking agent known for its ability to form strong hydrogen bonds with PVA [14,15]. Tannic acid also possesses inherent antimicrobial properties [16].

In this paper, we investigate the synthesis of curcumin-loaded organosolv lignin nanoparticles (Lig-Cur NPs) using continuous flow chemistry and their subsequent incorporation into a PVA solution, which is further crosslinked with tannic acid (TA) to enhance the material’s properties. The sol–gel transition achieved through a freeze–thaw process enables the formation of an antimicrobial hydrogel scaffold with a robust microporous structure. Here, we report the synthesis, characterization, and evaluation of this novel sponge, highlighting its potential as an effective solution for managing exudate and preventing bacterial colonization in surgical environments.

## 2. Results and Discussion

### 2.1. Synthesis and Characterization of Organosolv Lignin–Curcumin Nanoparticles (Lig-Cur NPs)

#### 2.1.1. Synthesis of Lig-Cur NPs Using Continuous Flow Chemistry

Curcumin is a hydrophobic molecule with a logP value ranging from 2.3 to 3.2 [17]. At neutral pH and room temperature, curcumin is insoluble in water; however, it dissolves well in polar solvents such as ethanol, dimethyl sulfoxide, and acetonitrile [18]. Organosolv lignin also exhibits solubility in ethanol while being insoluble in water. Therefore, ethanol was chosen as the solvent for the synthesis of lignin–curcumin nanoparticles, as it can effectively dissolve both curcumin and organosolv lignin. The water acted as a nonsolvent for these compounds. Consequently, a solvent-shifting strategy was employed using an ethanol/water solvent/nonsolvent system. Upon mixing organosolv lignin and curcumin in absolute ethanol and subsequently adding water, rapid nanoparticle formation occurred due to the non-covalent interactions such as the molecular self-assembly of the hydrophobic moieties (phenolic groups) of lignin and curcumin, hydrogen bonding interactions, and π-π interactions.

**Scheme 1:**
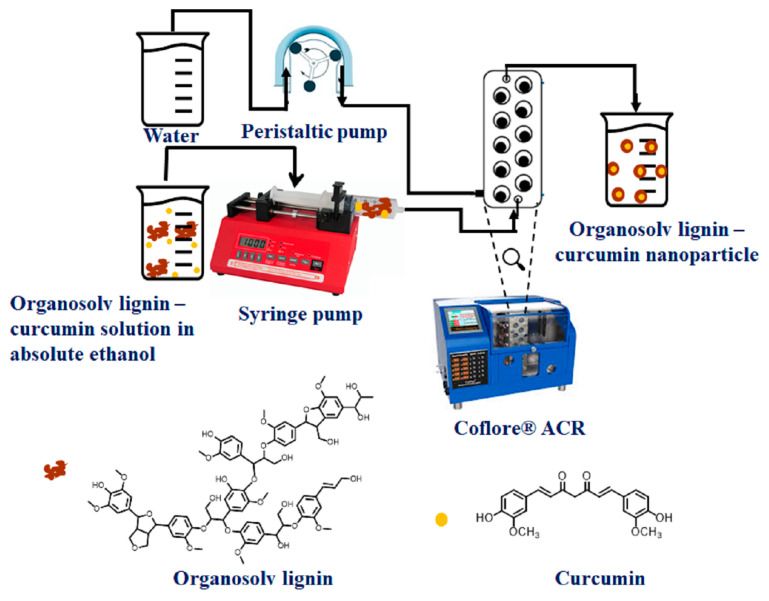
Schematic representation of the synthesis of organosolv lignin–curcumin nanoparticles in a continuous flow reactor.

The application of a continuous flow reactor in this synthesis process allowed for precise control over key parameters, such as the flow rate of organosolv lignin and water, as well as agitation. The plug-flow nature of the agitated cell reactor (ACR) ensured the continuous mixing of the ethanol solution of organosolv lignin and curcumin with the nonsolvent water, promoting efficient nanoparticle formation while minimizing the backflow issues often encountered in batch synthesis processes. This capability aids in fine-tuning the properties of the resultant nanoparticles. As a result, Lig-Cur NPs with a uniform size distribution and well-defined surface morphology were produced. The composition of the colloidal suspension of Lig-Cur Nps was 1 mg/mL of lignin and 9.8 μg/mL of curcumin. A schematic representation of this synthesis is shown in Scheme 1.

#### 2.1.2. Hydrodynamic Size and Surface Morphology of Lig-Cur NPs

The prepared Lig-Cur NPs were further characterized for their physicochemical properties by analyzing their hydrodynamic diameter, polydispersity index, and zeta potential using dynamic light scattering (DLS), as well as surface morphology through a Focus ion beam scanning electron microscope (FIB-SEM: Helios Nanolab 650 from FEI company, Hillsboro, OR, USA) and transmission electron microscopy (TEM: JEOL JEM-F200 cold FEG microscope, Tokyo, Japan). DLS analysis revealed that the average hydrodynamic diameter of Lig-Cur Nps is approximately 141 ± 6 nm, with a polydispersity index (PDI) of 0.2 (Table 1). This indicates that the nanoparticles exhibit homogeneity, which is a critical factor for several biological applications. In a similar study, Fontana et al. synthesized kraft lignin–curcumin nanoparticles by solvent shifting in the THF/water solvent/nonsolvent system. They reported that a high curcumin-to-lignin feed ratio, specifically 1 mg of curcumin to 10 mg of lignin, resulted in a larger hydrodynamic size of 387 ± 61 nm and a polydispersity index of 0.399 ± 0.059 [19]. In contrast, our study yielded a lower hydrodynamic size and PDI, which could be attributed to the differences in the type of lignin and solvents used. Additionally, we utilized a continuous flow reactor for the synthesis, which may have facilitated the production of less polydisperse nanoparticles. Furthermore, the zeta potential measurements indicated that the nanoparticles possessed a negative surface charge of −45 ± 6 mV, demonstrating very good colloidal stability due to the electrostatic repulsion among the Lig-Cur Nps (Table 1).

**Figure 1:**
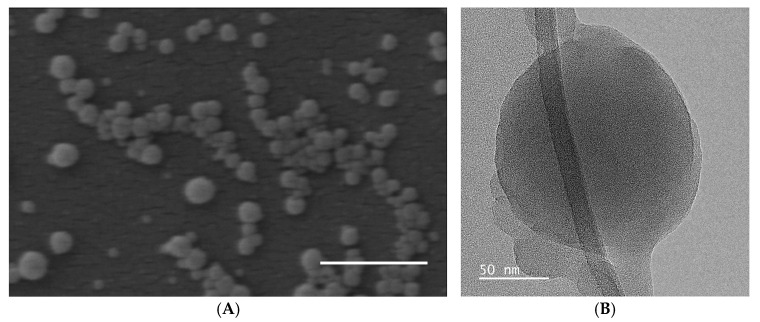
(**A**) Scanning electron microscopy imaging (scale bar is 500 nm) and (**B**) transmission electron microscopy imaging (scale bar is 50 nm) of Lig-Cur NPs.

The scanning electron microscopy (SEM) imaging (Figure 1A) of Lig-Cur NPs shows that the nanoparticles exhibited a spherical morphology with a dry particle diameter ranging from 100 to 150 nm. Transmission electron microscopy (TEM) analysis (Figure 1B) further reveals that the surface of the Lig-Cur Nps had a smooth and amorphous structure.

#### 2.1.3. Structural Characterization of Lig-Cur NPs Using Fourier Transform Infrared Spectroscopy

**Figure 2:**
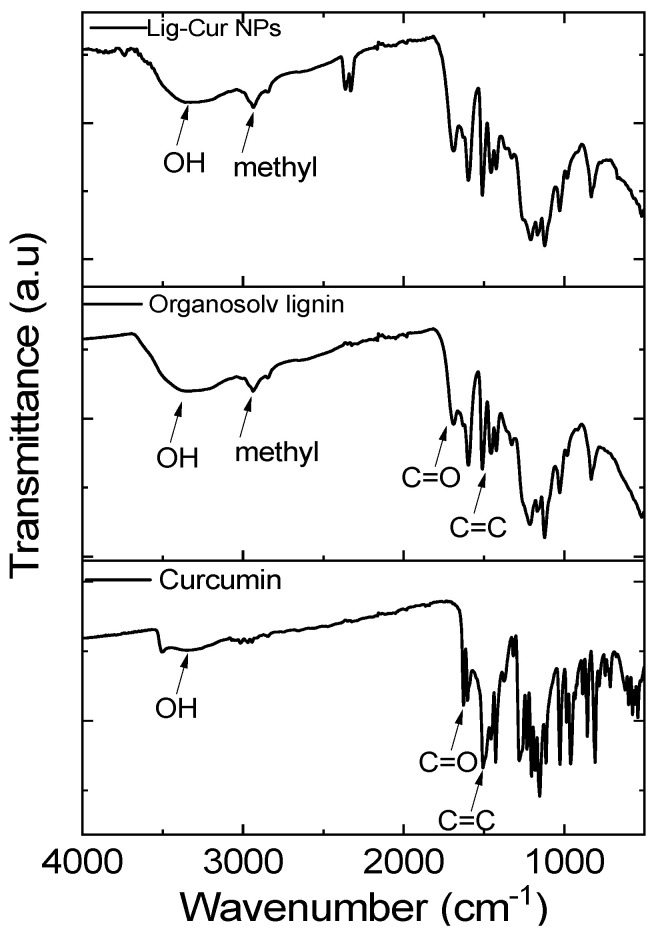
FTIR spectra of curcumin, organosolv lignin, and organosolv lignin-curcumin nanoparticles (Lig-Cur NPs).

The FTIR spectra of curcumin, organosolv lignin, and organosolv lignin–curcumin nanoparticles are shown in Figure 2. The characteristic bands of curcumin are as follows: the broad absorption band observed in the range of 3200–3540 cm^−1^ indicates the presence of hydroxyl (-OH) groups. The peaks at 1623 cm^−1^ and 1600 cm^−1^ are attributed to the diketone structure (C=O) of curcumin. The peaks in the region of 1505–1420 cm^−1^ correspond to C=C bonds. The peaks in the range of 1028–1282 cm^−1^ correspond to C-O stretching, indicating the presence of an ether bond in the curcumin structure [20].

The characteristic bands of organosolv lignin are as follows: the broad band in the region of 3689–3000 cm^−1^ is attributed to -OH stretching. The peaks at 2941 cm^−1^ and 2846 cm^−1^ are attributed to the symmetrical and asymmetrical vibrations of methyl and methylene groups. The peak at 1700 cm^−1^ is assigned to the C=O groups. The peaks in the range of 1593–1517 cm^−1^ are assigned to C=C stretching within the aromatic ring [21].

The FTIR spectrum of lignin–curcumin nanoparticles (Lig-Cur NPs) is very similar to that of organosolv lignin. This similarity may be due to the encapsulation of curcumin within the organosolv lignin nanoparticles or the overlap of characteristic bands from both curcumin and organosolv lignin, making it difficult to distinguish the differences in the spectrum.

### 2.2. Synthesis and Characterization of Tannic Acid-Crosslinked Polyvinyl Alcohol–Lig-Cur NPs with Hydrogel Composite Scaffolds (PVA-Lig-Cur-TA)

#### 2.2.1. Synthesis of PVA-Lig-Cur-TA Hydrogel Scaffolds

**Scheme 2:**
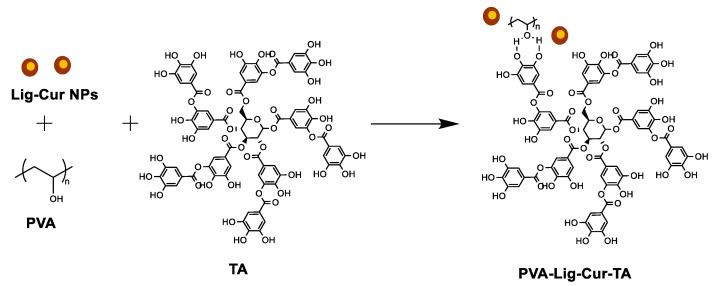
Synthesis of PVA-Lig-Cur-TA hydrogel scaffold.

The hydrogel composite scaffolds were successfully fabricated using polyvinyl alcohol (PVA), lignin-curcumin nanoparticles (Lig-Cur NPs), and tannic acid (TA) as the crosslinking agent. A schematic representation illustrating the synthesis of the PVA-Lig-Cur-TA scaffold is shown in Scheme 2.

**Figure 3:**
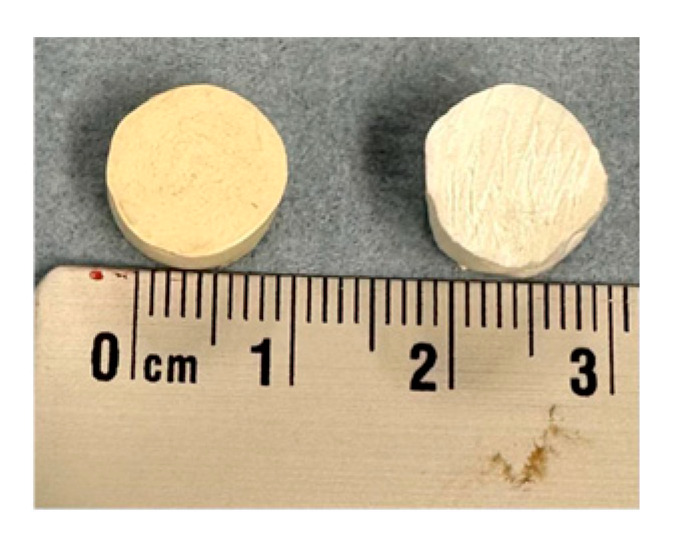
Photographs of PVA-Lig-Cur-TA scaffold (**left**) and PVA-TA scaffold (**right**).

PVA was dissolved in the Lig-Cur NP suspension in deionized water at a temperature of 70–75 °C while continuously stirring to ensure the complete dissolution of PVA and the uniform dispersion of the Lig-Cur NPs. This method facilitated the incorporation of Lig-Cur NPs into the PVA matrix, enhancing the potential bioactivity of the hydrogel scaffold. After the nanoparticles were incorporated, a tannic acid solution in deionized water was slowly added to the PVA-Lig-Cur NPs dispersion. Tannic acid is a polyphenolic compound composed of five digallic acid units linked to a glucose core and is rich in hydroxyl groups. Its high density of hydrogen-bonding donor sites, combined with a relatively elevated pKa of 8.5, leads to hydrogen-bonded molecular self-assembly that is more stable than PVA alone. It has been reported that the addition of TA to PVA can introduce stronger hydrogen bonding interactions between PVA and TA and a weaker hydrogen bonding interaction between PVA chains [22,23]. In this context, the addition of tannic acid in the PVA-Lig-Cur NPs suspension introduced multiple hydrogen bonding sites that interacted with both PVA and Lig-Cur NPs. In our study, the weight ratio of PVA to tannic acid was 40:1. The lower weight ratio of TA, along with continuous stirring and the dropwise addition of the TA solution, helped to prevent rapid hydrogen bonding interactions and the formation of gel particles during the process of addition. The concentrations of individual components in the PVA-Lig-Cur-TA formulation are given as follows: PVA at 80 mg/mL, lignin at 0.8 mg/mL, curcumin at 0.00784 mg/mL, and tannic acid at 2 mg/mL. The resulting mixture was then transferred into a mold and frozen to stabilize the scaffold structure. Lyophilization was employed to remove water from the system, yielding a porous and hydrogel scaffold. The combination of PVA, lignin–curcumin nanoparticles, and tannic acid produced a stable, bioactive hydrogel scaffold with potential biomedical applications. The presence of lignin, curcumin, and tannic acid within the scaffold may have further enhanced its antimicrobial properties, making it an ideal candidate for wound healing and other biomedical applications. A reference scaffold was prepared using polyvinyl alcohol and tannic acid (the PVA:TA weight ratio was 40:1) using the same method (Figure 3).

#### 2.2.2. Characterization of PVA and PVA-Lig-Cur-TA Scaffold by FTIR and XRD

**Figure 4:**
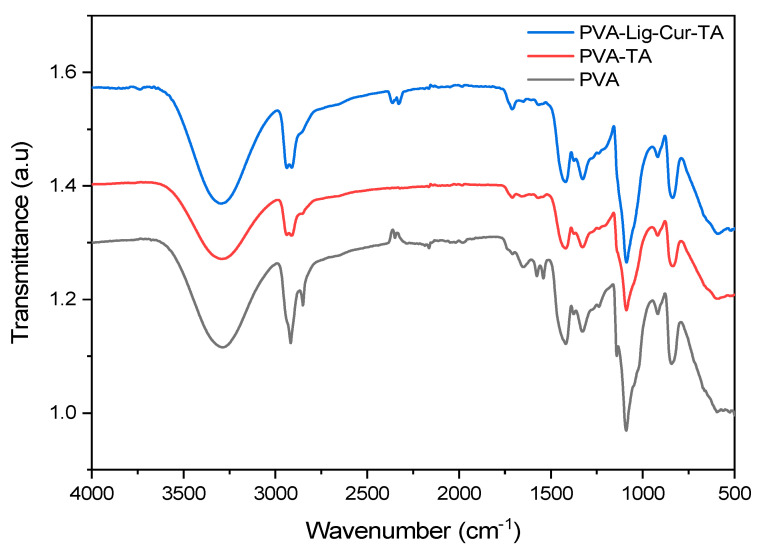
FTIR spectra of PVA alone, PVA-TA, and PVA-Lig-Cur-TA scaffold.

The FTIR spectra of PVA, PVA-TA, and PVA-Lig-Cur-TA scaffolds are shown in Figure 4. The FTIR spectrum of PVA exhibits its characteristic peaks, such as -OH stretching (3285 cm^−1^), C-H stretching (2800–3000 cm^−1^), and C-O (1000–1150 cm^−1^) bonds. In the PVA-TA scaffold, a new peak appears at 1709 cm^−1^, corresponding to the C=O stretching of the ester groups of tannic acid, indicating the incorporation of tannic acid. In the PVA-Lig-Cur-TA scaffold, in addition to the characteristic peaks of PVA-TA, the intensity of the peak at 1709 cm^−1^ increased, which is attributed to the C=O stretching of curcumin or changes due to the incorporation of Lig-Cur NPs [24].

**Figure 5:**
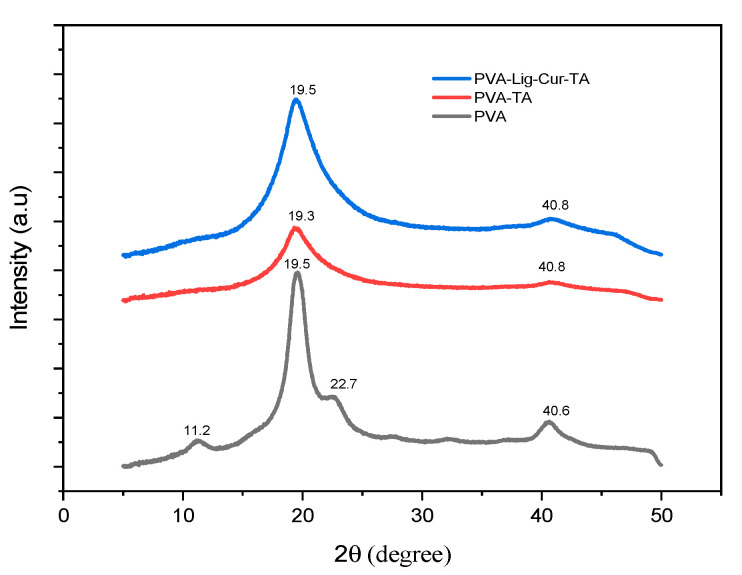
XRD pattern of PVA (powder), PVA-TA scaffold, and PVA-Lig-Cur-TA scaffold.

X-ray diffraction (XRD) was used to investigate the structure of PVA, PVA-TA, and PVA-Lig-Cur-TA scaffolds. As shown in Figure 5, four peaks were observed at 2θ = 11.2°, 19.5°, 22.7°, and 40.6° for pure PVA, which can be assigned to the (001), (101), (200), and (102) planes of PVA crystallites. It is widely recognized that PVA exhibits crystallinity as a result of the strong intermolecular interactions between PVA chains through hydrogen bonding [22]. For the PVA–TA scaffold, the XRD pattern displayed peaks at 2θ = 19.3° and 40.8° with lower intensity compared to pure PVA, indicating a decrease in crystallinity. The strong hydrogen bonding interactions between PVA and TA may have interrupted the alignment of PVA chains. The PVA-Lig-Cur-TA scaffold also exhibited peaks at 2θ = 19.5° and 40.8° with higher intensity compared to PVA-TA [23]. The Lig-Cur NPs can form hydrogen bonding interactions with tannic acid, which may limit the hydrogen bonding interactions with PVA. Additionally, Lig-Cur NPs may have acted as a barrier, reducing the interaction between PVA and TA.

#### 2.2.3. Surface Morphology of PVA-Lig-Cur-TA Hydrogel Scaffolds

**Figure 6:**
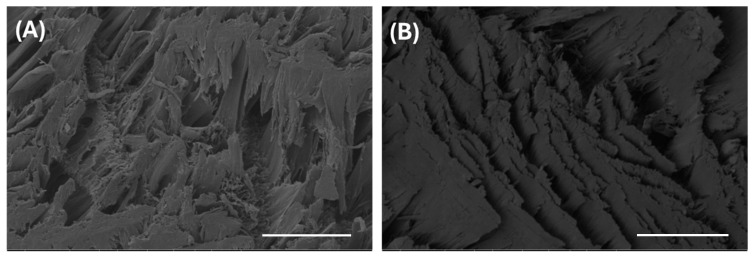
Scanning electron microscopy images of (**A**) tannic acid-crosslinked polyvinyl alcohol–lignin–curcumin composite hydrogel scaffold and (**B**) tannic acid-crosslinked polyvinyl alcohol scaffold, scale bar is 100 µM.

The microstructures of the PVA-TA scaffold and PVA-Lig-Cur-TA scaffold (Figure 6) were observed using focused ion beam scanning electron microscopy (SEM). The horizontal scaffold sections were cut using a scalpel blade. The surface morphology of the PVA–TA and PVA-Lig-Cur-TA scaffolds demonstrated a maximum pore size of 20 μm.

**Figure 7:**
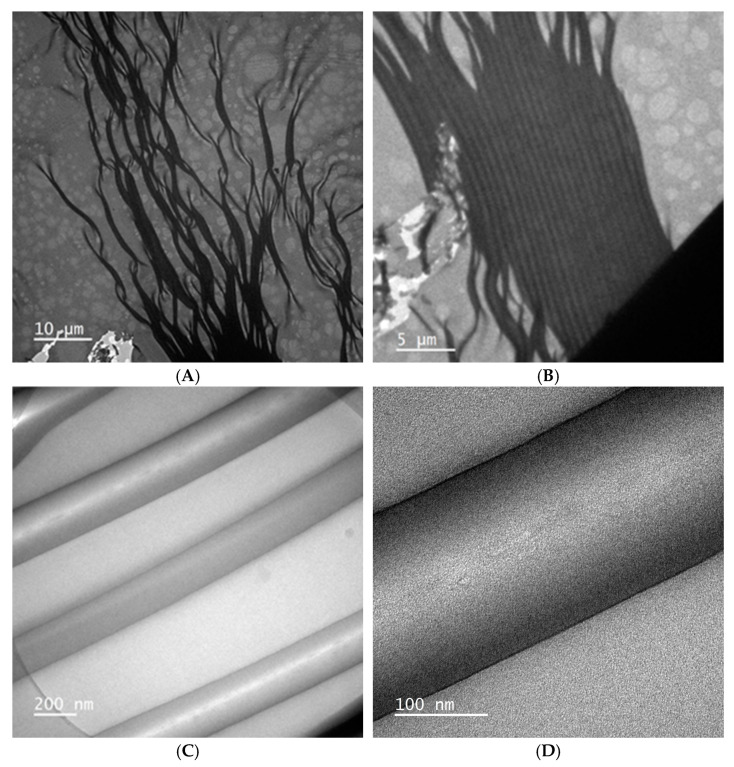
Transmission electron microscopy images of cryo-sectioned PVA-Lig-Cur-TA scaffold imaged at different magnifications. (**A**) Low magnification image showing the fibrous texture. (**B**) Individual fibers aligned to form a crystalline domain (**C**) Higher magnification image of individual fibers. (**D**) Amorphous surface morphology of the individual fiber.

The PVA-Lig-Cur-TA scaffold cryosections were imaged with a transmission electron microscope. The cross-section revealed a fibrous morphology with an approximate fiber diameter of 200 nm (Figure 7). Individual fibers forming a crystalline domain are shown in Figure 7B. The amount of TA added to the PVA-Lig-Cur-TA formulation was not sufficient to alter the semi-crystalline nature of PVA to amorphous state. Zooming into Figure 7B–D, a smooth nanofiber with amorphous morphology on the surface is displayed. As described in the preceding Section 2.1.2, Lig-Cur NPs also exhibit amorphous morphology; consequently, the presence of Lig-Cur NPs could not be differentiated from the PVA bulk in the TEM image of the PVA-Lig-Cur-TA cryosection.

#### 2.2.4. Swelling and Degradation

**Figure 8:**
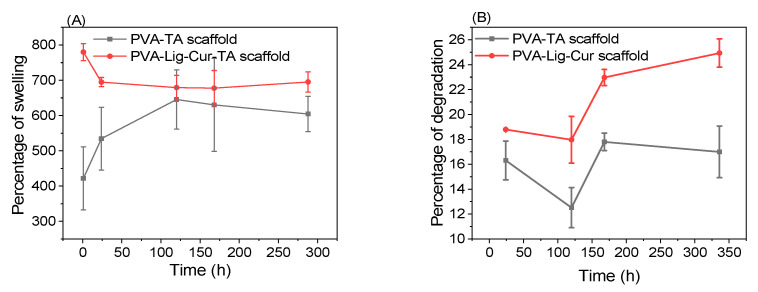
Percentage of (**A**) swelling and (**B**) degradation of the PVA-TA scaffold and the PVA-Lig-Cur-TA scaffold.

Both the PVA-TA scaffold and the PVA-Lig-Cur-TA scaffold used in this study were crosslinked with tannic acid. Both scaffolds exhibited rapid liquid adsorption, reaching a maximum at 150 h, after which the swelling gradually decreased. In the PVA-Lig-Cur-TA scaffold, swelling remained relatively stable throughout the study, indicating consistent water retention properties (Figure 8A). The enhanced stability in the PVA-Lig-Cur-TA scaffold may be attributed to the interactions between PVA, lignin, curcumin, and the crosslinking agent tannic acid.

The degradation in this study demonstrates that both the PVA-TA scaffold and PVA-Lig-Cur-TA scaffold show variations, initially decreasing in degradation up to 100 h and then increasing after 150 h (Figure 8B). This behavior could be due to the dissolution or surface erosion of non-crosslinked polyvinyl alcohol present in the scaffold. The PVA-Lig-Cur-TA scaffold showed higher degradation than the PVA scaffold, suggesting that incorporating Lig-Cur NPs may reduce the interaction between PVA and the crosslinking agent tannic acid, thereby enhancing the surface erosion of free polymer chains. After degradation studies, the water used for scaffold immersion was dried, and 1 mL of ethanol was added. This solution was analyzed using an infinite M1000 Pro UV-Visible absorption spectrophotometer (Tecan Austria GmbH, Grödig, Austria) at a wavelength of 420 nm (water was used as a reference) to determine if any curcumin was released from the scaffold. However, no release of curcumin was observed after 350 h of incubation in Milli-Q water.

#### 2.2.5. Thermal Stability

**Figure 9:**
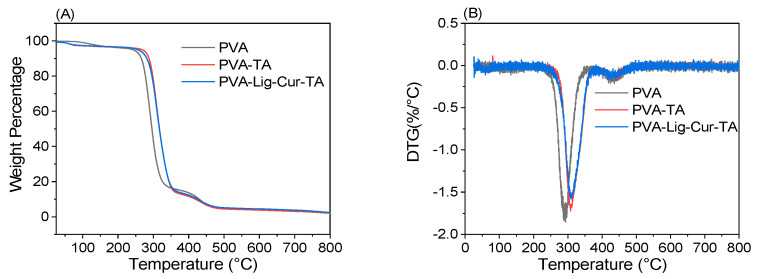
(**A**) Thermogravimetric analysis and (**B**) differential thermal analysis of pure PVA, PVA-TA scaffold, and PVA-Lig-Cur-TA scaffold.

The TGA thermograms of pure PVA, the PVA-TA scaffold, and the PVA-Lig-Cur-TA scaffold are shown in Figure 9. Pure PVA exhibits three regions of weight loss. The first region, from 30 to 200 °C (4% weight loss), is attributed to the loss of bound water. The second region, from 225 to 343 °C (80% weight loss), correlates with the decomposition of PVA side chains. The third region, from 343 to 480 °C (11.3% weight loss), is associated with the decomposition of the PVA backbone. After 500 °C, the small weight loss (less than 2%) was due to charred carbon.

The thermograms of PVA-TA and PVA-Lig-Cur-TA exhibited nearly identical thermal degradation profiles. Both thermograms display a three-step degradation pattern. Similarly to the pure PVA thermogram, the first region of weight loss occurs between 30 and 200 °C (4% weight loss) and is due to the loss of adsorbed water. The second degradation stage, from 200 to 350 °C (80% weight loss), is attributed to the decomposition of PVA side chains and PVA crosslinked with tannic acid. The third region, from 365 to 480 °C (9.4% weight loss), is associated with the decomposition of the PVA backbone. The onset of degradation for pure PVA is 294 °C, while that for PVA-TA and PVA-Lig-Cur-TA is 314 °C. This increase in the onset of degradation for PVA-TA and PVA-Lig-Cur-TA can be attributed to the formation of a stable network resulting from crosslinking with tannic acid.

#### 2.2.6. Antimicrobial Testing on the Scaffold

PVA-Lig-Cur-TA scaffold specimens have demonstrated significant antibacterial activity against *Staphylococcus aureus* and *Pseudomonas aeruginosa*. According to ISO 20743 [25], the efficacy of the antibacterial properties was evaluated with a 1.7 log reduction against *Staphylococcus aureus* (for the difference in bacterial growth between the PVA-Lig-Cur-TA scaffold and PVA-TA scaffold) and a 6.2 log reduction against *Pseudomonas aeruginosa*, respectively (Table 2). Notably, the growth of *Staphylococcus aureus* after 24 h of incubation was lower in the PVA-Lig-Cur-TA scaffold compared to the control specimens (PVA-TA). Interestingly, the antibacterial effect of PVA-Lig-Cur-TA was significantly greater against the Gram-negative microorganism *Pseudomonas aeruginosa*. In a direct comparison to PVA-TA control specimens, the growth of *P. aeruginosa* was completely inhibited within 24 h of incubation in the PVA-Lig-Cur-TA scaffolds. Furthermore, no viable P. aeruginosa microorganisms were detected after this incubation period, highlighting the efficacy of the scaffold. This strong antibacterial performance may be attributed to the combined effects of lignin, curcumin, and tannic acid within the scaffold. Curcumin possesses broad-spectrum antibacterial actions, allowing it to target resistant strains, including those responsible for surgical-related infections, such as *Staphylococcus aureus* and Escherichia coli [26,27]. Tannic acid is also demonstrated to be active against *Staphylococcus aureus* and Staphylococcus epidermidis [28]. Morena et al. synthesized phenolated lignin nanoparticles using lignin and tannic acid and observed that these nanoparticles are effective against both Gram-positive and Gram-negative bacteria [29]. The multifunctional nature of the PVA-Lig-Cur-TA scaffolds suggests not only the presence of antimicrobial and bacteriostatic properties but also their potential for use in biomedical applications where infection control is paramount. Such characteristics make these scaffolds promising candidates for wound dressing applications, where the prevention of bacterial colonization is critical for successful recovery.

### 2.3. Further Discussion

The development of PVA-Lig-Cur-TA scaffolds represents a significant advancement in biobased and biocompatible materials for wound dressing systems. The incorporation of curcumin, known for its anti-inflammatory and antioxidant properties, alongside organosolv lignin, a natural polymer with antimicrobial characteristics, creates a nanocomposite scaffold that enhances biological performance. The observed antibacterial activity in PVA-Lig-Cur-TA scaffolds, particularly against *Staphylococcus aureus* and *Pseudomonas aeruginosa*, highlights the potential of these materials to combat bacterial infections. The 1.7 log reduction in *Staphylococcus aureus* and 6.2 log reduction in *Pseudomonas aeruginosa* bacterial growth, as assessed by ISO 20743, confirms that the scaffold functions not only as a matrix for the absorption of exudate but also actively inhibits bacterial proliferation. This dual functionality is crucial in applications where infection control is paramount, such as in post-surgical cases and wound dressing. Moreover, the combination of curcumin and organosolv lignin may offer synergistic effects in promoting healing and reducing inflammation. Importantly, the consideration of biocompatibility is essential to ensure that the scaffold provides a suitable environment for tissue regeneration. Thus, further studies are required to investigate the biocompatibility and long-term performance of these scaffolds in various wound environments, particularly across different wound types and sizes.

## 3. Conclusions

In this study, we successfully developed a novel PVA-Lig-Cur-TA scaffold that addresses the critical need for materials with both high absorption and antimicrobial properties for managing exudate in post-surgical applications. By synthesizing curcumin-loaded organosolv lignin nanoparticles (Lig-Cur NPs) through a solvent shifting approach and incorporating it into polyvinyl alcohol (PVA) and crosslinking it with tannic acid, we created a unique nanocomposite scaffold. This hydrogel scaffold exhibits an interconnected microporous network, achieving a remarkable swelling percentage of 800%, which facilitates the rapid absorption of exudate. Furthermore, the scaffold demonstrates antimicrobial and bacteriostatic properties against *Staphylococcus aureus* and *Pseudomonas aeruginosa*. The PVA-Lig-Cur-TA hydrogel exerts a more potent effect on *P. aeruginosa*. *S. aureus* and *P. aeruginosa* are responsible for a large proportion of hospital-acquired and healthcare-associated infections in patients and are potentially multi-resistant to antibiotic drugs. This result highlights the ability of these scaffolds to prevent bacterial colonization while effectively managing wound fluid. These findings suggest that PVA-Lig-Cur-TA scaffolds represent a promising option for the management and prevention of bacterial colonization in surgical settings. Future studies should further explore the long-term performance and biocompatibility of this scaffold to validate its potential as a standard material for advanced wound dressing applications.

## 4. Materials and Methods

### 4.1. Materials

Polyvinyl alcohol (EXCEVAL™ RS-2117) was kindly provided by Kuraray Poval, Frankfurt am Main, Germany. Organosolv lignin was purchased from Chemicalpoint UG, Oberhaching, Germany. Absolute ethanol was acquired from VWR, Fontenay-Sous-Bois Cedex, France. Curcumin (≥94% curcuminoid content, ≥80% curcumin content) was purchased from Sigma-Aldrich, Overijse, Belgium. Tannic acid (Source: Chinese natural gall nuts) was purchased from Sigma-Aldrich. Deionized water was obtained by filtering water through a Milli-Q Ultrapure water purification system using a 0.2 µm PES high flux capsule filter with 18.2 MΩ·cm ionic purity at 23 °C in the laboratory.

### 4.2. Methods

#### 4.2.1. Synthesis of Organosolv Lignin–Curcumin Nanoparticles (Lig-Cur Nps)

We dissolved 0.5 g of organosolv lignin in 100 mL of absolute ethanol in a 250 mL round bottom flask. We stirred the mixture at room temperature for 30 min to ensure complete dissolution. Then, curcumin (4.90 mg) was added to this organosolv lignin solution, and the solution was stirred for another 30 min to promote the interaction between lignin and curcumin. Then, this organosolv lignin–curcumin solution was taken in a 50 mL glass syringe and mixed with deionized water in a continuous flow reactor. The process parameters for the synthesis are the flow rate of organosolv lignin at 10 mL/min, the flow rate of water at 50 mL/min, and agitation at 9 Hz in room-temperature conditions. The organic solvent, ethanol, was removed using a rotary evaporator, and the recovered nanoparticles were stored in a glass container at room temperature in the dark for further studies.

#### 4.2.2. Fabrication of Polyvinyl Alcohol–Lig-Cur Nps–Tannic Acid Composite Scaffold (PVA-Lig-Cur-TA)

PVA (2 g) was dissolved in 20 mL of the Lig-Cur NPs suspension by heating at 70 °C for 4 h. Then, 50 mg of tannic acid was dissolved in 5 mL of deionized (DI) water, which was added dropwise to the PVA-Lig-Cur NPs dispersion while stirring the mixture at 70 °C. Stirring was continued for another 15 min, after which the mixture was transferred into a plastic mold and frozen at −20 °C. Finally, it was freeze-dried to obtain the PVA-Lig-Cur-TA composite scaffold.

#### 4.2.3. Fabrication of Tannic Acid-Crosslinked Polyvinyl Alcohol (PVA-TA) Scaffold

PVA (2 g) was dissolved in 20 mL of deionized water by heating at 70 °C for 4 h. Then, 50 mg of tannic acid was dissolved in 5 mL deionized (DI) water and added dropwise to the PVA solution while stirring the mixture at 70 °C. Stirring was continued for another 15 min, after which the mixture was transferred into a plastic mold and frozen at −20 °C. Finally, it was freeze-dried to obtain the tannic acid-crosslinked PVA scaffold. This scaffold was used as a reference scaffold for further studies.

#### 4.2.4. Structural Characterization by Fourier Transform Infrared Spectroscopy (FTIR) and X-Ray Diffraction (XRD) Analysis

The Fourier transform infrared spectra were obtained using a Bruker Vertex 70 spectrophotometer with an attenuated total reflection (ATR) accessory in the wavelength range of 4000–500 cm^−1^ over 50 scans, with a resolution of 1 cm^−1^. The XRD measurements were performed on a Panalytical X’Pert Pro XRD equipped with a Cu anode in reflection geometry.

#### 4.2.5. Particle Size and Zeta Potential Analysis Using Dynamic Light Scattering

The hydrodynamic particle size distribution, polydispersity index (PDI), and zeta potential of Lig-Cur NPs suspensions were measured with Malvern Zetasizer Nano-ZS90 equipment (UK). Before measuring the hydrodynamic particle size distribution, colloidal suspensions were diluted in water, and 1 mL was transferred to a polystyrene cuvette. The samples were equilibrated in the analysis chamber for 120 s before measuring the backscatter angle (173°) at 25 °C. The zeta potential measurements were performed using a Malvern ZETA potential capillary cell (DTS1070). The samples were analyzed in triplicate, with 12–15 measurements per replication.

#### 4.2.6. Scanning Electron Microscopy (SEM)

The surface morphology of Lig-Cur Nps was characterized using focus ion beam scanning electron microscopy (FIB SEM HELIOS NANOLAB 650, FEI company, USA). The diluted Lig-Cur Nps was deposited on a silicon wafer and dried at room temperature; then, they were sputter-coated with gold (7 nm). The horizontal and vertical cross-sections of the PVA-Lig-Cur Nps composite scaffold and PVA-TA reference scaffold were attached on a carbon tape, sputter-coated with gold (7 nm), and analyzed usingFIB SEM HELIOS NANOLAB 650, FEI company.

#### 4.2.7. Transmission Electron Microscopy (TEM)

Transmission electron microscopy analysis were performed on a JEOL JEM-F200 cold FEG microscope operating at an acceleration voltage of 200 kV. The Lig-Cur nanoparticles were deposited on a holey carbon grid (400 Mesh copper). The PVA-Lig-Cur-TA scaffold was covered with cryo resin, ratified using a cryo-ultramicrotome, and collected on a holey carbon grid for imaging.

#### 4.2.8. Swelling and Degradation Studies

The percentages of swelling and degradation of the PVA-Lig-Cur-TA composite scaffold and PVA-TA reference scaffold were measured gravimetrically. Briefly, square-shaped scaffolds were immersed in milliQ water under static conditions. At different time points, water-absorbed samples were taken out, and reweighed. The swelling percentage was calculated according to Equation (1).

Swelling (%) = ((W_t_ − W_0_)/W_0_) 100 (1)

where W_0_ is the initial mass of the dried sample, and W_t_ is the mass of the swollen sample at a given time point t.

The degradation analysis was performed in deionized water at 37 °C. At different time points, water-soaked samples were taken out, freeze-dried and reweighed. The percentage of degradation was calculated following Equation (2):

Degradation (%) = ((W_0_ − W_tdry_)/W_0_) 100 (2)

where W_0_ is the initial mass of the dried sample, and W_tdry_ is the mass of the freeze-dried sample at a given time point t.

#### 4.2.9. Thermogravimetric Analysis

The thermal stability of PVA, PVA-TA, and PVA-Lig-Cur-TA scaffolds was assessed using a thermogravimetric analyzer (TGA 2, Mettler Toledo GmbH, Giesen, Germany). For the analysis, 10 mg of each sample was placed in an alumina pan and heated from 25 °C to 800 °C at a heating rate of 10 °C/min under a nitrogen atmosphere at a flow rate of 90 mL/min.

#### 4.2.10. Antimicrobial Testing

The antimicrobial activity of PVA-Lig-Cur-TA scaffold specimens was performed using the absorption method described in the European Standard EN ISO 20743:2021 [25], “Determination of antibacterial activity of textile products” with minor modifications. An inoculation loop (Culti-Loops™ *Staphylococcus aureus* subsp. *aureus* ATCC™ 6538™, ThermoFischer Diagnostic NV (Zaventem, Belgium), R4607016 or Culti-Loops™ *Pseudomonas aeruginosa* ATCC™ 15442™, ThermoFischer Scientific, R4607210) was used and spread on a Tryptone Soy Agar (TSA, ThermoFischer Scientific PO5012A) plate, which was then incubated at 37 °C for 24 h. After incubation, a single colony was harvested using a sterile inoculation loop and incubated in an Erlenmeyer flask containing 20 mL of Nutrient Broth (NB) at 37 °C under constant agitation (100 rpm) for 18 to 24 h.

A second culture was initiated from this initial culture by adding 0.4 mL of inoculum containing 1 × 10^8^ CFU/mL to a final concentration of 3 × 10^8^ CFU/mL in an Erlenmeyer flask with 20 mL of fresh NB medium. The Erlenmeyer flask was then incubated at 37 °C with constant agitation (100 rpm) until a bacterial concentration of approximately 10^7^ CFU/mL was reached. To prepare the test inoculum, the bacterial concentration was adjusted spectrophotometrically to a concentration ranging from 1 × 10^5^ CFU/mL to 3 × 10^5^ CFU/mL in 20-fold diluted Tryptone Soy Broth (TSB).

Six specimens of the PVA-Lig-Cur-TA composite scaffold (treated specimens) and six specimens of the non-treated scaffold, i.e., the PVA-TA scaffold (control specimens), were inoculated by depositing 100 μL of the test inoculum at different points on each specimen until complete absorption was achieved. Immediately after inoculation, 5 mL of the neutralization solution (Soya Casein Digest Lecithin Polysorbate Broth, SCDLP Broth) was added to three control specimens and three treated specimens. After vortexing, bacterial concentrations were assessed for each suspension using the plate counting method. The remaining specimens were incubated at 37 °C in tightly closed vials for 24 h. At the end of the incubation, the specimens were also immersed in 5 mL of the neutralization solution (SCDLP), and their bacterial concentration was assessed following the same procedure.

The efficiency of the antimicrobial activity was then calculated using the following equation:

A = (lg Ct – lg C0) − (lg Tt – lg T0)(3)

where A is the antibacterial activity value.

lg Ct and C0 are the common logarithms of the average number of bacteria obtained from three control specimens after 24 h of incubation and immediately after inoculation, respectively.

lg Tt and lg T0 are the common logarithms of the average number of bacteria obtained from three treated specimens after 24 h of incubation and immediately after inoculation, respectively.

## Figures and Tables

**Table 1:** Characteristics of Lig-Cur NPs.

Nanoparticles	Hydrodynamic Diameter (nm)	Polydispersity Index (PDI)	Zeta Potential (mV)
Lig-Cur NPs	141 ± 6	0.2	−45 ± 6

**Table 2:** Antimicrobial test results (log colony forming unit, CFU).

Strain Designation	Gram Stain	Specimens (n = 3)	Contact Time = 0 h	After 24 h Incubation	Growth Value	Antibacterial Activity Value (Log10 CFU)
S. aureus ATCC 6538	Gram-positive	Control PVA-TA	C0 (Log10 CFU)	Ct (Log10 CFU)	Ct-C0 (Log10 CFU)	1.7
			4.6	6.9	2.3	
		PVA-Lig-Cur-TA	T0 (Log10 CFU)	Tt (Log10 CFU)	Tt-T0 (Log10 CFU)	
			4.2	4.8	0.6	
P. aeruginosa ATCC 15442	Gram-negative	Control PVA-TA	C0 (Log10 CFU)	Ct (Log10 CFU)	Ct-C0 (Log10 CFU)	>6.2
			3.3	6.3	3	
		PVA-Lig-Cur-TA	T0 (Log10 CFU)	Tt (Log10 CFU)	Tt-T0 (Log10 CFU)	
			3.2	<0.1	<−3.2	

## Data Availability

Data can be obtained from the corresponding author upon reasonable request.
